# Evaluating the small-scale epidemiology of the stony-coral -tissue-loss-disease in the middle Florida Keys

**DOI:** 10.1371/journal.pone.0241871

**Published:** 2020-11-11

**Authors:** William C. Sharp, Colin P. Shea, Kerry E. Maxwell, Erinn M. Muller, John H. Hunt

**Affiliations:** 1 Fish and Wildlife Research Institute, Florida Fish and Wildlife Conservation Commission, Marathon, Florida, United States of America; 2 Fish and Wildlife Research Institute, Florida Fish and Wildlife Conservation Commission, St. Petersburg, Florida, United States of America; 3 Mote Marine Laboratory & Aquarium, Sarasota, Florida, United States of America; Biodiversity Research Center, TAIWAN

## Abstract

Along the Florida reef tract, stony-coral-tissue-loss disease (SCTLD) has caused extensive mortality of more than 20 scleractinian coral species. The pathogen is unknown, but its epizoology indicates that the disease, facilitated by water currents, has progressed linearly along the tract, affecting reefs at the scale of hundreds of kilometers. To inform ongoing disease mitigation efforts, we examined the small-scale spatial and temporal epidemiology of SCTLD. We established a series of sites in the middle Florida Keys at offshore and inshore locations that had not yet shown signs of SCTLD. We then conducted high-frequency monitoring from February 2018 through September 2019 and documented the onset of SCTLD and its progression through the sites. SCTLD was first observed at one site during early February 2018 and by early March 2018 all sites showed signs of the disease. A dynamic multistate model suggested that disease transmission was independent of coral density and found little evidence of a positive association between a colony showing signs of SCTLD and the condition or distance to its neighboring colonies. The model did, however, indicate that the probability of a colony showing signs of SCTLD increased with increasing colony surface area. These results are consistent with the water-borne transmission of a pathogen that progressed rapidly through the survey area. However, by the end of our survey the progression of SCTLD had slowed, particularly at inshore sites. Many affected colonies no longer exhibited progressive tissue mortality typical of the disease, suggesting the existence of differentially resilient colonies or coral communities, meriting their use for future coral rescue and propagation and disease research. These results are useful for refining ongoing SCTLD mitigation strategies, particularly by determining when disease rates are sufficiently low for direct intervention efforts designed to arrest disease progression on individual coral colonies will be most effective.

## Introduction

Disease can play a major role in structuring marine ecosystems and has been recognized as a driving force behind declines in reef-building corals worldwide leading to cascading effects throughout these ecosystems [1, 2]. Extensive effort has been directed at assessing the pathology, etiology, epizoology of specific diseases [3] and understanding the mode of transmission of these diseases, which may be spread through a water-borne or vector-borne pathogen, via direct contact, or most often, a combination of these modes [4–6], and is particularly vital to inform the development of management strategies to mitigate the effects of epizootics [7]. However, assessing the potential modes of disease transmission can often prove challenging due to the complex etiologies of different coral diseases [8]. Examining the spatial epidemiology and temporal dynamics of these events can provide insight into the disease ecology and mode of transmission [9].

The Caribbean region has been described as a “hotspot” for coral disease [3], and the occurrence of virulent short-lived epizootics has become increasingly common. As of 2000 nearly 70% of the world’s described coral disease occurred with this region [10]. The first reports of coral disease along the Florida reef tract (FRT) emerged in the 1970s, and numerous diseases have since been documented with increasing frequency [11]. The (FRT) is now experiencing the most widespread and virulent coral disease outbreak on record. This disease, termed stony-coral-tissue-loss disease (SCTLD), was first reported near Miami, FL, in 2014 [12], and by 2019 it had affected coral communities from the northernmost extent of the FRT to Key West, FL. The disease affects at least 20 species of scleractinian corals, including primary reef builders and species listed as threatened under the Endangered Species Act, though it has exhibited pronounced species-specific prevalence rates [12]. A case definition has been developed that describes the visual appearance of SCTLD [13]. In brief, the morphology of SCTLD is generally described as focal or multifocal lesions exhibiting acute or subacute areas of tissue loss, resulting in patches of stark white areas of newly denuded skeleton, though signs can vary across species. The disease differs from previous diseases documented along the FRT by its unprecedented continuous–and now multi-year–prevalence that has resulted in significant declines in the abundance of susceptible species on affected reefs which has likely altered ecosystem function [14]. Diseased coral colonies exhibiting signs consistent with SCTLD have recently been reported within reefs in Mexico, Jamaica, Belize, the Dominican Republic, St. Maarten, the US Virgin Islands, the Turks and Caicos Islands, and Sint Eustatius [15, 16].

At present, the pathogen(s) responsible for SCTLD has not been identified, but a consortium of bacteria, most of which have been previously associated with other coral diseases, has been detected in association with SCTLD [17]. Moreover, the application of antibiotics to affected coral colonies, both in the laboratory and in the field, has been demonstrated to slow or stop the disease’s progression [18], further suggesting that bacterial pathogens are associated with SCTLD. Nevertheless, viral or eukaryotic cells may also be involved [19]. Broadscale spatial epidemiological models have shown that the progression of SCTLD along the FRT was consistent with waterborne transmission and was positively associated with high coral diversity [9].

The severity of the SCTLD epidemic has resulted in what is perhaps an unprecedented concerted effort involving numerous researchers and resource managers throughout south Florida to coordinate a response and develop a management strategy to slow or prevent the spread of SCTLD [20]. A concerted effort is underway to identify and accurately diagnose the causative etiological agent(s) and its modes and rate of transmission, document its distribution and prevalence, identify any contributing environmental factors, and develop novel approaches to mitigating disease progression at the colony level [18, 21]. An array of mitigation strategies has been proposed including treating coral colonies exhibiting signs of SCTLD through direct application of disinfectants, antibiotics, or probiotics, amputating the affected areas of diseased colonies, and targeting diseased or highly susceptible species for removal [22]. Unfortunately, because SCTLD is so virulent and has progressed so broadly and rapidly along the FRT, information about its small-scale transmission in coral communities fundamental to the development of a mitigation strategy has been lacking.

In early 2018, we initiated a localized, cross-reef, high-frequency monitoring effort to assess the small-scale spatial epidemiology of SCTLD with the goal of informing resource managers developing a disease response strategy to mitigate the effects of this disease along the FRT, particularly considering colony-specific intervention efforts. Here, we summarize monitoring efforts conducted from January 2018 through mid-September 2019. We first describe the coral communities at a series of “sentinel sites” established in an area of the middle Florida Keys where SCTLD had yet to be observed, then document both the cross-reef and within-reef progression of SCTLD and species-specific mortality rates at these sites. We then summarize a predictive model of spatially and temporally explicit disease-transmission dynamics at those sites, discuss possible environmental and ecological factors driving the observed spatial and temporal dynamics, and the implications of our findings to the ongoing effort to address this epizootic.

## Materials and methods

### Sentinel site selection and establishment

**Fig 1:**
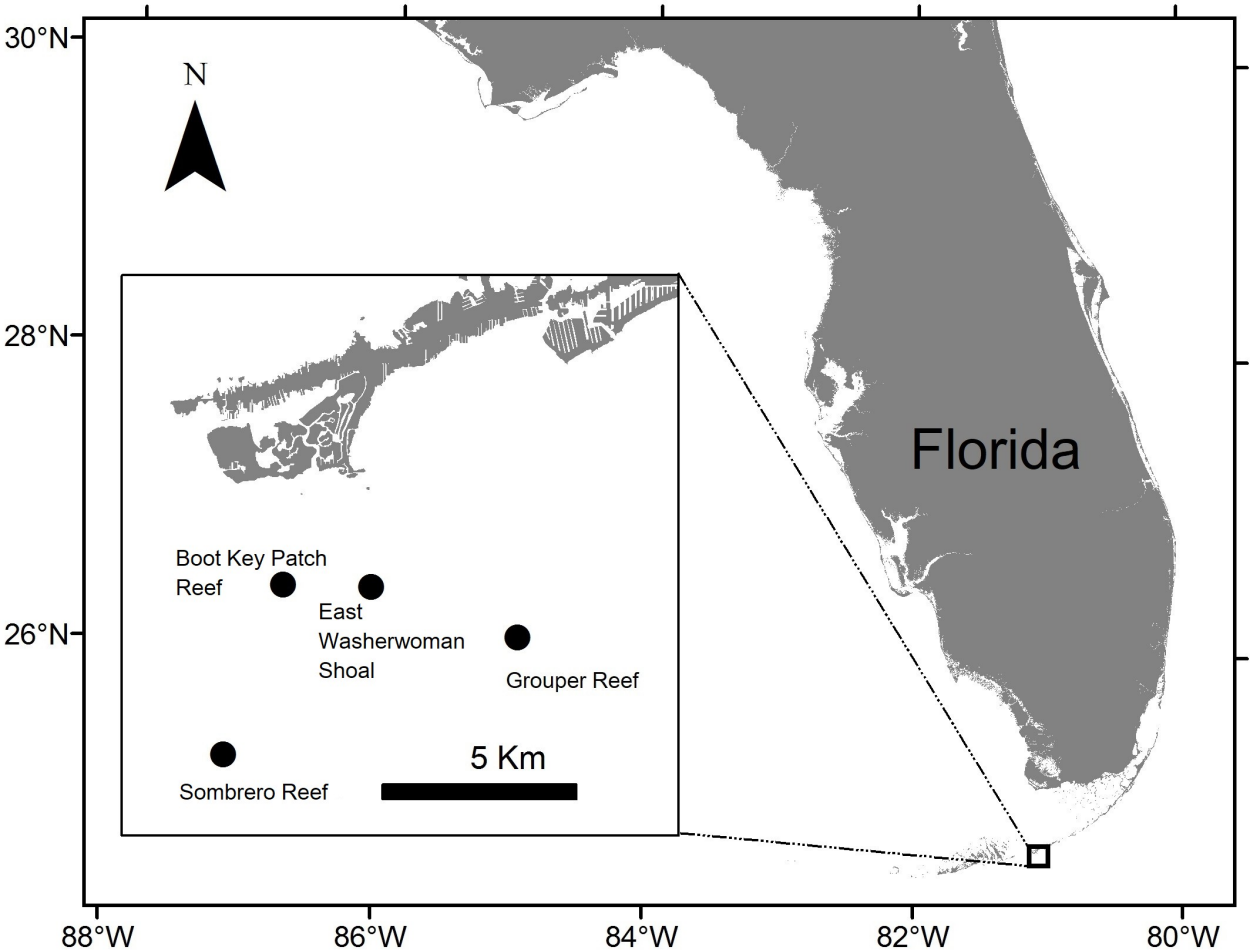
Location of the four sentinel sites. The Boot Key Patch Reef and East Washerwoman Shoal sites are patch reefs located shoreward of the offshore bank reef system. The Grouper Reef and Sombrero Reef sites are located on offshore bank reef habitat.

By October 2017, extensive coral mortality associated with SCTLD had progressed from the origination area off the coast of Miami southwestward along the FRT to Long Key in the middle Florida Keys. Anticipating that SCTLD would continue to progress southwesterly along the FRT, we established four sentinel reef sites off Marathon, approximately 20 km southwest of Long Key during January 2018. Roving diver surveys of these sites confirmed that the coral community did not exhibit signs of SCTLD at that time. Two sentinel sites were selected within inshore patch reef habitat (East Washerwoman Shoal, 24°39′51.34″N, 81°4′25.86″W, and an unnamed patch reef (herein referred to as Boot Key Patch Reef, 24°39′53.60″N, 81°5′46.18″W), and two were located on offshore reef habitat (Sombrero Reef, 24°37′31.30″N, 81°6′41.04″W, and Grouper Reef, 24°39′9.25″N, 81°2′11.47″W) (Fig 1). At each site we established two replicate plots. The replicate plots at the East Washerwoman Shoal, Boot Key Harbor patch reef, and Sombrero Reef sites were 12–17 m apart; those at Grouper Reef were 38m apart. The water depth at each plot was ~7 m. We targeted the study area of the two plots at each location based on the density and species diversity of corals, in particular those species of boulder corals described as especially susceptible to the disease (e.g., *Dichocoenia stokesii*, *Meandrina meandrites*, *Pseudodiploria strigosa*, and *Colpophyllia natans*, *Montastraea cavernosa*, *Orbicella faveolata*) [13]. Both replicate plots at each location were of the same size, but plot size differed among sites. Our goal was to establish plots that were representative of the coral communities at each location yet of an area that made it feasible for divers to locate and assess the disease status of each coral colony greater than 10 cm diameter at a plot. Therefore, at each inshore location we established two 25-m^2^ (5 m × 5 m) plots. At the offshore locations, coral was less dense than at the inshore locations, so the plots at Sombrero Reef each measured 49-m^2^ (7 m– 7 m) each, and those at Grouper Reef measured 100-m^2^ (10 m– 10 m) each.

To establish a plot, two divers each extended a surveyor’s tape along the substrate at the appropriate distance (e.g., 5 m for a 25-m^2^ plot or 10 m for a 100-m^2^ plot) with the tapes at a 90° angle to each other. The divers swam diagonally the length of the hypotenuse of the tapes, and using Pythagoreans’ Theorem, ensured that the two sides were square and installed nails to mark two sides of the plot. The remaining two sides of the plot were then established. Divers secured marked plastic tags to the substrate with a nail at one-meter intervals on the north and south sides of the plot. The nails securing the tags facilitated the attachment of surveyor’s tapes at 1-m intervals across the plot to orient divers within the plot and allowed them to record the relative position of each coral colony within the plot as detailed below.

Once the plots had been established, we conducted a baseline survey of the coral community at each plot using methods similar to those of the Florida Reef Resilience Program’s Disturbance Response Monitoring for shallow coral reefs in the Florida Keys [23]. Divers identified and recorded each coral colony >10 cm at its greatest diameter within a plot to the lowest practicable taxonomic level; they also included a few colonies <10 cm of species previously observed to be highly susceptible to SCTLD. Each coral colony width, length, and height were measured to the nearest 0.1 cm, and their location with respect to the north/south and east/west axes of the plot was recorded (±0.1 m). Divers then recorded the proportion of living coral tissue and the proportion of exposed skeleton (often colonized by algae or other encrusting organisms) due to previous, presumably non-SCTLD related mortality events for each colony.

We calculated the surface area (SA) of each coral colony by treating it as an idealized hemi-ellipsoid and, using our three size measurements of colony size (width, length, and height), applied the equation of Thomsen to our three size measurements to approximate its SA [14].

Our equation was:

$$\begin{array}{c} \mathrm{SA}\approx {2\pi (\frac{{ab}^{1.6075}+{ac}^{1.6075}+{bc}^{1.6075}}{3})}^{1/1.6075}\\ a=\mathrm{colony}\;\mathrm{height}\\ b=\mathrm{colony}\;\mathrm{width}/2\\ c=\mathrm{colony}\;\mathrm{length}/2 \end{array}$$

Using this calculation, the area of living coral tissue per colony would be estimated using the following formula

AreaofLivingTissue=SA(1–(%OriginalMortalty+%SCTLD−RelatedMortality))

### Monitoring the within- and across-reef spatial and temporal progression of SCTLD

**Fig 2:**
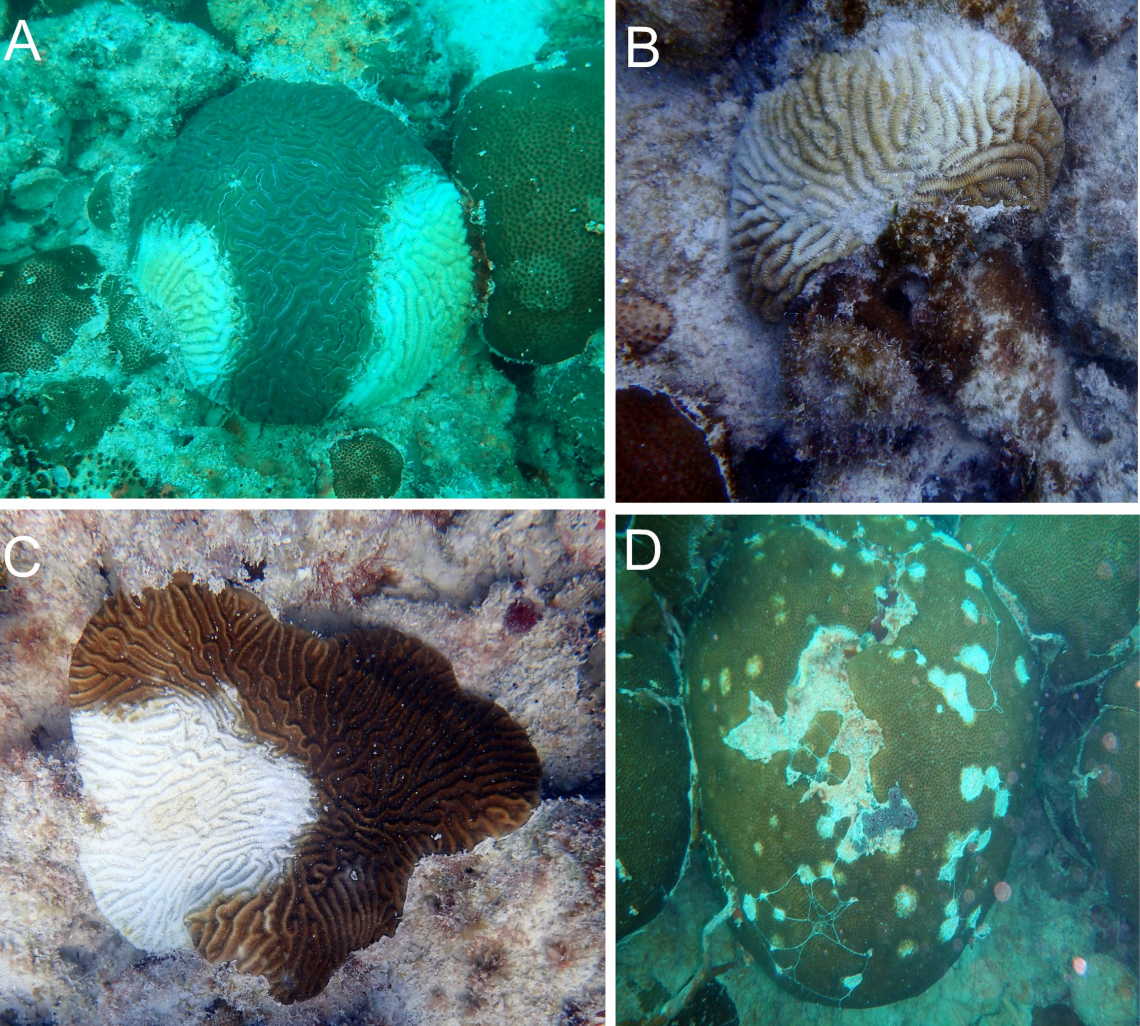
Photographs of SCTLD-affected coral colonies. Coral colonies showing signs of SCTLD. (A) *Diploria labyrinthiformis*. (B) *M*. *meandrites*. (C) *P*. *strigosa*. (D) *Siderastrea siderea*.

Following our baseline assessments, both plots at each site were revisited approximately every two weeks February 2018 through mid-August 2018 and then monthly from late August 2018 through September 2019. Using the position of each colony recorded during the baseline surveys, we created maps of each plot to assist divers in relocating each colony during subsequent surveys. Using these maps during each monitoring period, all previously identified coral colonies were located and assessed visually for signs of SCTLD as described in the case definition that details the species-specific visual appearance of SCTLD [13]. If a colony exhibited signs of SCTLD as per the case definition, the affected portion of the colony was recorded as a percentage of whole colony surface area (Fig 2). Divers also carried data listing all coral colonies being monitored and for each condition observed during the previous survey (i.e., the proportion of the colony with living tissue, the proportion exhibiting older mortality recorded during the baseline survey, and the proportion affected by SCTLD, if any) to assist them in estimating the proportion of the colony affected by SCTLD during each survey. The colony was also visually assessed for bleaching. We recorded bleaching status as a categorical variable that indicated either: i) no bleaching, ii) paling, iii) partly bleached, and iv) fully bleached.

### Nearest-neighbor analysis and multistate disease-transmission model

Our primary modeling objective was to identify the predominant factors influencing temporal changes in the status of coral colonies (i.e., non-diseased, diseased, dead). Because the relative locations of all coral colonies to each other were known, we first calculated the Euclidean distances between all pairs of colonies in each plot at each site using the R package ‘spatstat’ [24]. We then determined, for each colony, the distance to its nearest neighbor, which remained constant for all monitoring periods. In addition to distance, we determined the status of each colony’s nearest neighboring colony during each monitoring period.

Using the observation histories associated with each coral colony for the full duration of the study, we developed a 3-state dynamic-occupancy model for each reef [25]. For the initial survey at time t, we modeled the occupancy state of each colony as a categorical random variable with one of three possible values *z*: alive and not diseased (i.e., no signs of SCTLD) (z = 1); alive and diseased (i.e., showing signs of SCTLD) (z = 2); and dead (z = 3). For subsequent surveys, transitions between occupancy states were defined by a transition-probability matrix to determine the probability of a colony being in each possible state at time t + 1 given its state at time t. The state-transition probabilities were further defined by processes of infection (γ) and survival of non-diseased (Φ_ND_) and diseased (Φ_D_) colonies associated with each transition. For example, if a non-diseased colony remained non-diseased between times t and t + 1, it would be described as having transitioned from z = 1 to z = 1 during that time. This transition is expressed mathematically as: P[z_t+1_ = 1|z_t_ = 1] = Φ_ND_*(1 − γ), where Φ_ND_ represents the probability of an uninfected colony surviving, and γ represents the probability of a colony being diseased given that the colony survived the interval (1 − γ represents the probability of a colony not becoming diseased). We assumed that the probability of a diseased colony becoming non-diseased was zero, which was consistent with our field observations (i.e., once colonies are diseased, colonies either died or remained chronically diseased). For a given interval, t to t + 1, the nine possible state transitions were represented by the following probabilistic statements:

1. P[z_t+1_ = 1|z_t_ = 1] = Φ_ND_*(1 − γ)
2. P[z_t+1_ = 2|z_t_ = 1] = Φ_ND_*γ
3. P[z_t+1_ = 3|z_t_ = 1] = (1 − Φ_ND_)*(1 - γ)
4. P[z_t+1_ = 1|z_t_ = 2] = 0
5. P[z_t+1_ = 2|z_t_ = 2] = Φ_D_
6. P[z_t+1_ = 3|z_t_ = 2] = (1 − Φ_D_)
7. P[z_t+1_ = 1|z_t_ = 3] = 0
8. P[z_t+1_ = 2|z_t_ = 3] = 0
9. P[z_t+1_ = 3|z_t_ = 3] = 1

We modeled each state-transition parameter using a logit link function and included several covariates that we hypothesized might influence each state transition parameter: colony surface area, distance to nearest neighbor, status of nearest neighbor (binary variables indicating dead, non-diseased, or diseased neighbors, where non-diseased served as the statistical baseline), and two interaction terms: distance to nearest neighbor × dead and distance to nearest neighbor × diseased. To facilitate model fitting and convergence, we standardized the distance to nearest neighbor and colony SA covariates to have a mean of zero and standard deviation of one; hence, parameter estimates were interpreted as a change in the log-odds of state-transition parameters for every unit (1 standard deviation) change in a continuous predictor variable. To account for possible dependence among species and time periods (i.e., colonies in these groups likely responded similarly to SCTLD), we also included a random intercept associated with survey period (γ only) and with species (Φ_D_ and γ). All random effects were assumed to be normally distributed with a mean of zero and random effect–specific variance [26]. We used Markov chain Monte Carlo (MCMC) simulation, as implemented in JAGS v 4.3.0, to fit the models [27]. All models were fitted by running three parallel chains, each with 20,000 iterations, a burn-in of 10,000 (i.e., the first 10,000 iterations were discarded), and diffuse priors. We assessed model convergence using the Gelman-Rubin diagnostic [28] and by examining trace plots for each model parameter. Finally, we conducted a posterior-predictive check to assess goodness-of-fit by simulating replicated data under each fitted model and comparing summary statistics from the replicated data to those of the observed data [18]. We calculated goodness-of-fit statistics as the ratio of total predicted to total observed number of coral colonies in each of the three occupancy states, where Bayesian p-values < 0.05 and > 0.95 indicated lack of fit.

#### Ethics statement

Activities in this study were conducted in accordance with the terms of research permit numbers FKNMS– 2018–006 and FKNMS– 20018–108 issued to the Florida Fish and Wildlife Conservation Commission by the National Oceanic and Atmospheric Administration, Office of National Marine Sanctuaries, Florida Keys National Marine Sanctuary.

## Results

### Monitoring the within- and across-reef spatial and temporal progression of SCTLD

#### Coral community

The baseline surveys of the coral communities at the four sentinel sites were conducted from January 10 through February 2, 2018, and no coral colonies exhibiting signs of SCTLD were observed. Excluding two colonies of the Acroporid coral *Acropora cervicornis*, which are not susceptible to SCTLD [29] and are not addressed here, we identified 1,341 coral colonies comprising 23 species across families (Table 1). Because the coral species composition and cover were similar between each of the replicate plots of each site, we have pooled the plots to facilitate visual assessment for the presentation and our general description of the sites.

**Table 1:** Coral species identified on at least one of the sentinel sites.

**Agariciidae**				
	*Agaricia agaricites* (Linnaeus 1758)			
**Astrocoeniidae**				
	*Stephanocoenia intersepta* (Lamarck 1816)			
**Faviidae**				
	*Colpophyllia natans* (Houttuyn 1772)			
	*Diploria labyrinthiformis* (Linnaeus 1758)			
	*Pseudodiploria clivosa* (Ellis & Solander 1786)			
	*Pseudodiploria strigosa* (Dana 1846)			
	*Scolymia* spp. (Haime 1852)			
**Meandrinidae**				
	*Meandrina jacksoni* (Pinzón 2011)			
	*Meandrina meandrites* (Linnaeus 1758)			
	*Dichocoenia stokesii* (Milne Edwards & Haime 1848)			
	*Eusmilia fastigiata* (Pallas 1766)			
**Merlinidae**				
	*Orbicella annularis* (Ellis & Solander 1786)			
	*Orbicella franksi* (Gregory 1895)			
	*Orbicella faveolata* (Ellis & Solander 1786)			
**Montastraeidae**				
	*Montastraea cavernosa* (Linnaeus 1767)			
**Mussidae**				
	*Mycetophyllia aliciae* (Wells 1973)			
	*Mycetophyllia* spp. (Milne Edwards & Haime 1848)			
**Oculinidae**				
	*Oculina diffusa* (Lamarck 1816)			
**Poritidae**				
	*Porites astreoides* (Lamarck 1816)			
	*Porites* (Pallas 1766)			
**Siderastreidae**				
	*Siderastrea radians* (Pallas 1766)			
	*Siderastrea siderea* (Ellis & Solander 1786)			
**Scleratinia inserta sedis**				
	*Solenastraea bournoni* (Milne Edwards 1849)			

**Fig 3:**
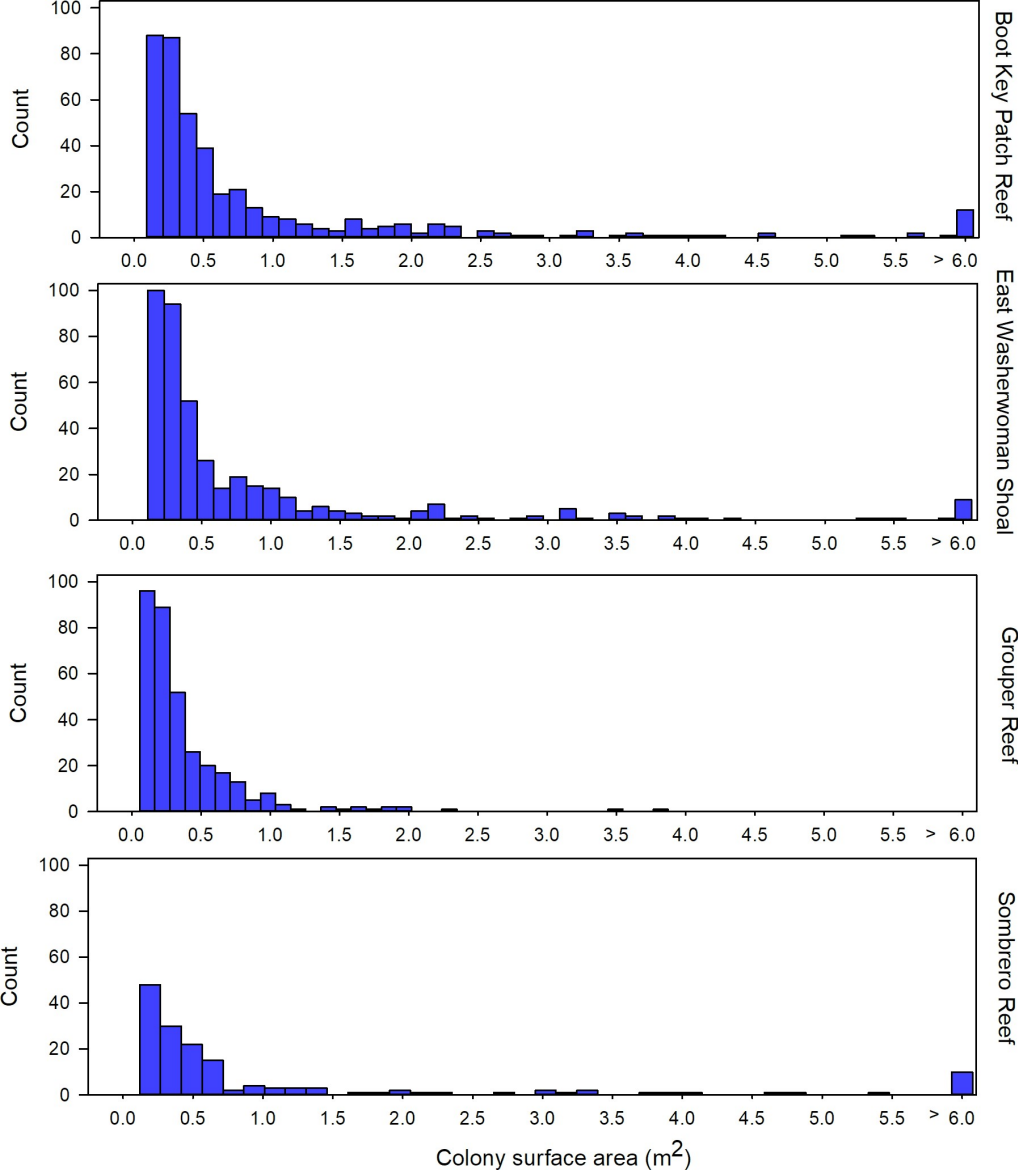
Size frequency histograms summarizing coral colony size as total skeletal surface area. Site-level coral community size structure estimated from the baseline surveys of the sentinel sites conducted from January 10 through February 2, 2018. Colony size structure estimated calculating SA for each colony. SA for each coral colony was derived using its width, length, and height measurements taken during the baseline surveys and calculating area as an idealized hemi-ellipsoid.

**Fig 4:**
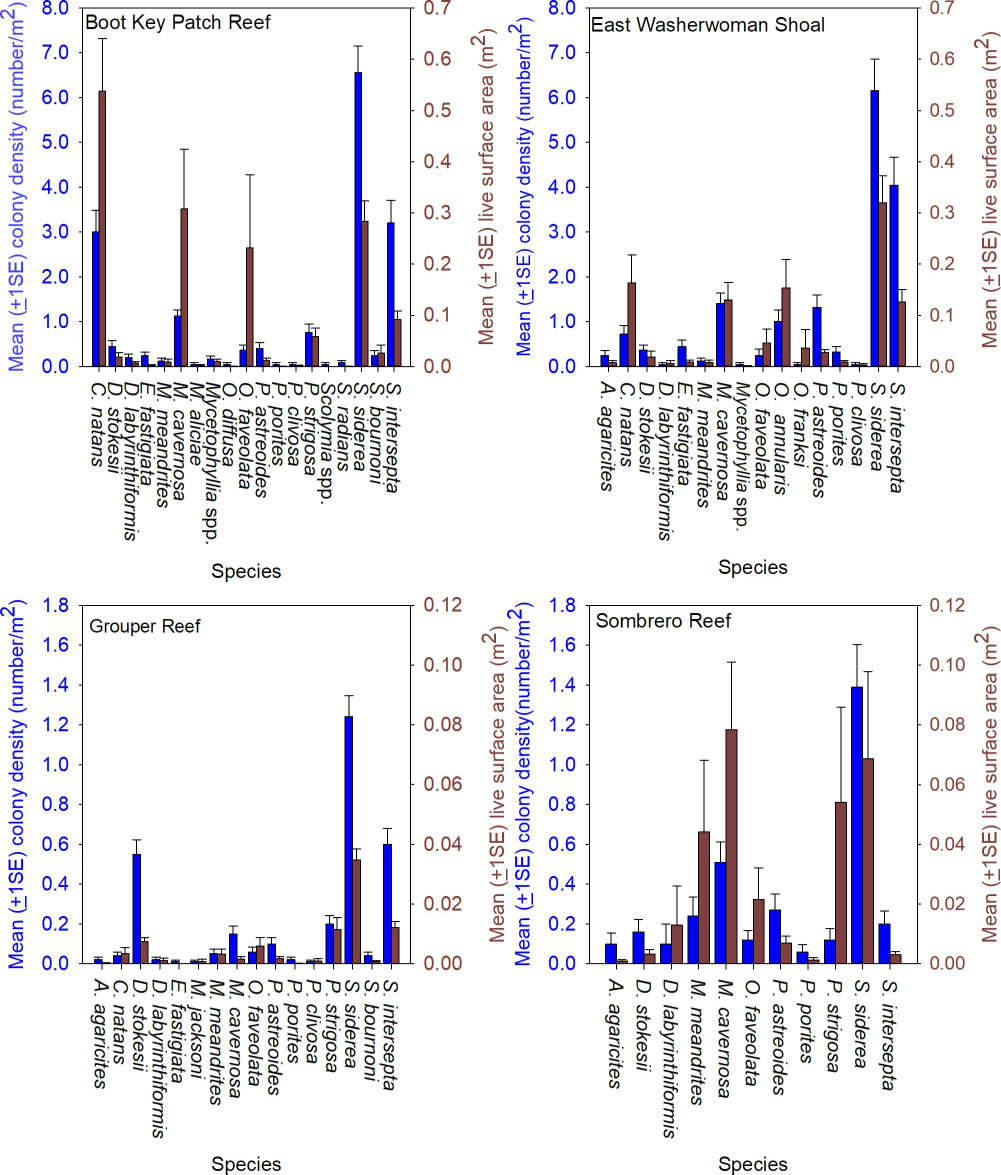
Coral community composition at the four sentinel sites. The mean (±1SE) number of coral colonies per m^2^ and mean (±1SE) living coral tissue (m^2^), per m^2^ at each of the four sentinel sites observed during the baseline surveys conducted January 10 through February 2, 2018. Living coral tissue was derived by first estimating SA for each coral colony using its width, length, and height measurements taken during the baseline surveys and calculating area as an idealized hemi-ellipsoid. The area of living tissue was then estimated from the proportion of the colony SA observed with of living tissue relative to the total colony area.

The coral community size structure was generally similar across the four sites, but more large colonies were present at the inshore sites, Boot Key Patch Reef and East Washerwoman, relative to the offshore sites Grouper Reef and Sombrero Reef (Fig 3). Sixteen species were identified on at least one offshore and one inshore site, and 10 species were common to all four sites, but the coral community at the inshore sites had a greater number of coral species and higher densities of living coral tissue compare to the offshore sites. Twenty-two species were present at the inshore sites. (Fig 4). Those two sites were dominated numerically by *Siderastrea siderea*, which accounted for 38% of colonies, followed by *Stephanocoenia intersepta* (22%), *Colpophyllia natans* (11%), *M*. *cavernosa* (8%), *Porites astreoides* (5%), *Orbicella annularis* (3%), *D*. *stokesii* (2%), and *P*. *strigosa* (2%). The overall SA of living tissue summed across all coral colonies was 39.94 m^2^ at Boot Key Patch Reef and 26.50 m^2^ at East Washerwoman Shoal, respectively. Six species accounted for approximately 90% of the living coral biomass: *C*. *natans* (26%) *S*. *siderea* (23%), *M*. *cavernosa* (16%), *O*. *faveolata* (10%), *S*. *intersepta* (8%), and *O*. *annularis* (6%).

Sixteen species were present at the offshore sites. The two offshore sites were largely dominated numerically by the same species as the inshore sites: *S*. *siderea* (41%), *S*. *intersepta* (15%), *D*. *stokesii* (14%), *M*. *cavernosa* (8%), *P*. *strigosa* (6%), *P*. *asteroids* (6%) and *M*. *meandrites* (4%). The estimated SA of living tissue summed across all coral colonies in at the two offshore sites was 9.43 m^2^ at Grouper Reef and 14.09 m^2^ at Sombrero Reef. Seven species accounted for approximately 93% of the living coral biomass: *S*. *intersepta* (31%), *M*. *cavernosa* (17%), *P*. *strigosa* (17%), *M*. *meandrites* (11%), *O*. *faveolata* (7%), *S*. *intersepta* (6%), and *D*. *stokesii* (4%).

#### SCTLD monitoring

By September 2019, the last monitoring period summarized, we had located 14 additional coral colonies that were not observed in the baseline assessment and had included them in in our monitoring effort. Including these colonies, we identified and monitored 1,355 colonies. We note that the total number of colonies assessed during each period differed slightly due to the addition of these colonies and because we were not always able to locate all previously located colonies. The difference was small however, the mean (± 1 sd) proportion of colonies located and surveyed at each site per monitoring period ranged from 97% to 99.7% (Boot Key, 99.7% ±0.32%; East Washerwoman Shoal, 99.7% ±0.35%; Grouper Reef, 97.0% ±2.46%; Sombrero Reef, 99.6% ±0.60%).

**Fig 5:**
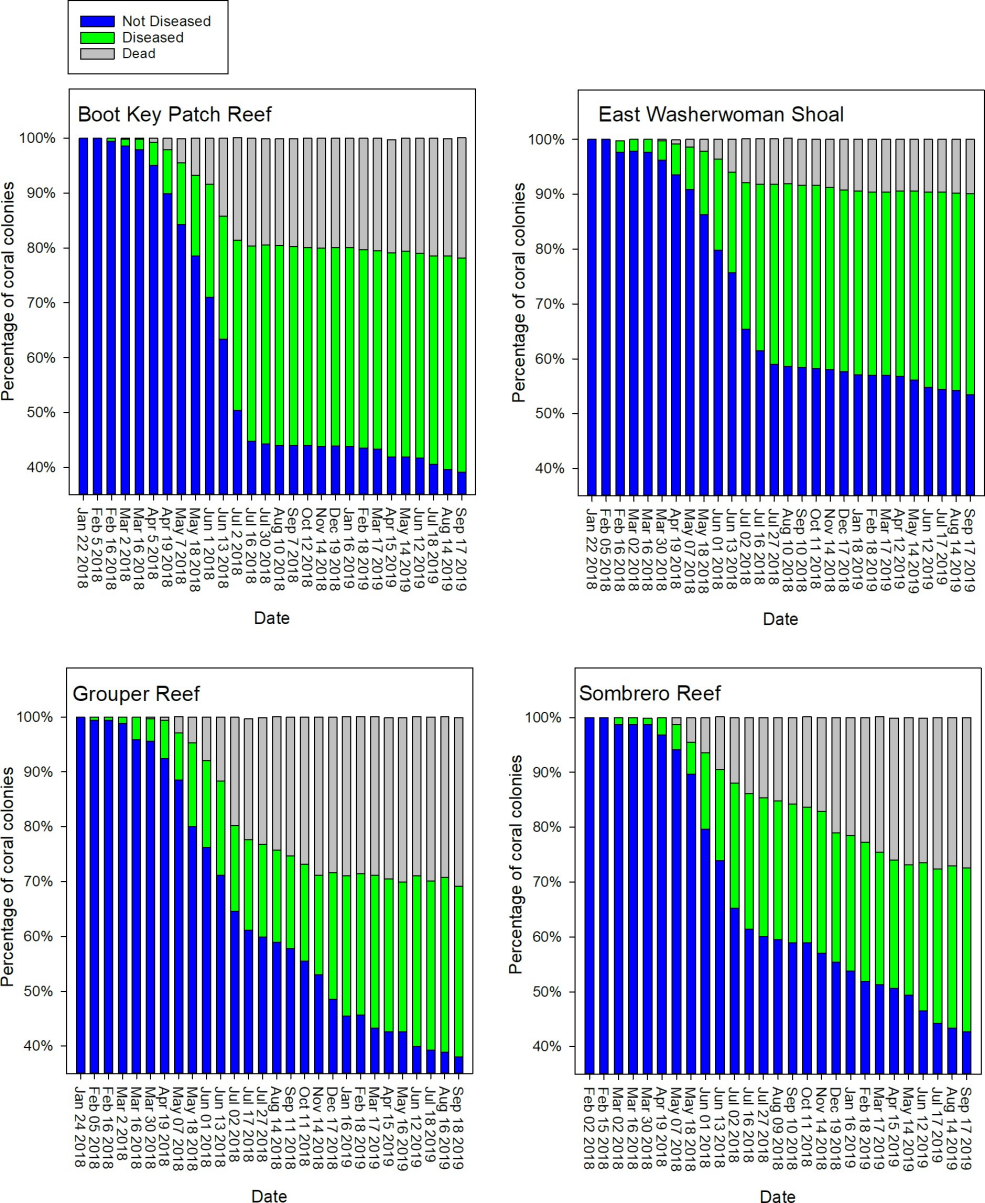
Time series of the disease status of coral colonies. Status is defined as: i) not diseased, ii) diseased, and iii) dead. Status is summarized as the percent of total colonies per site from late January 2018 through mid-September 2019. Observations were made bimonthly through July 2018 and monthly thereafter.

**Fig 6:**
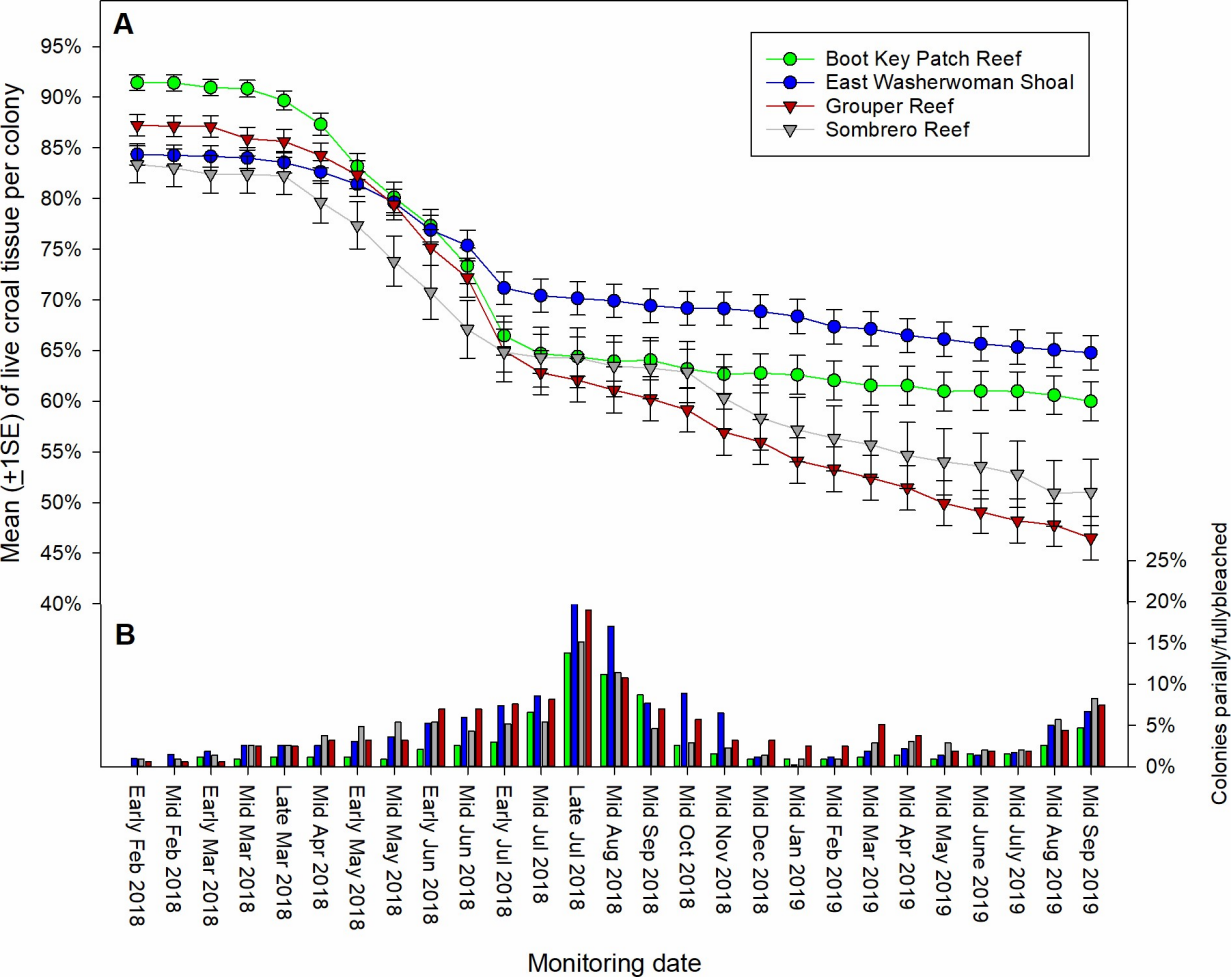
Time series summarizing living coral tissue and bleaching. (A) Mean (±1SE) percentage of living coral tissue per colony for each of the sentinel sites from early February 2018 (before SCTLD was observed) through September 2019 (replicate plots pooled). Observations were made bimonthly through July 2018 and monthly thereafter. (B) The percentage of coral colonies that were either partly or fully bleached by site over the same time frame.

**Fig 7:**
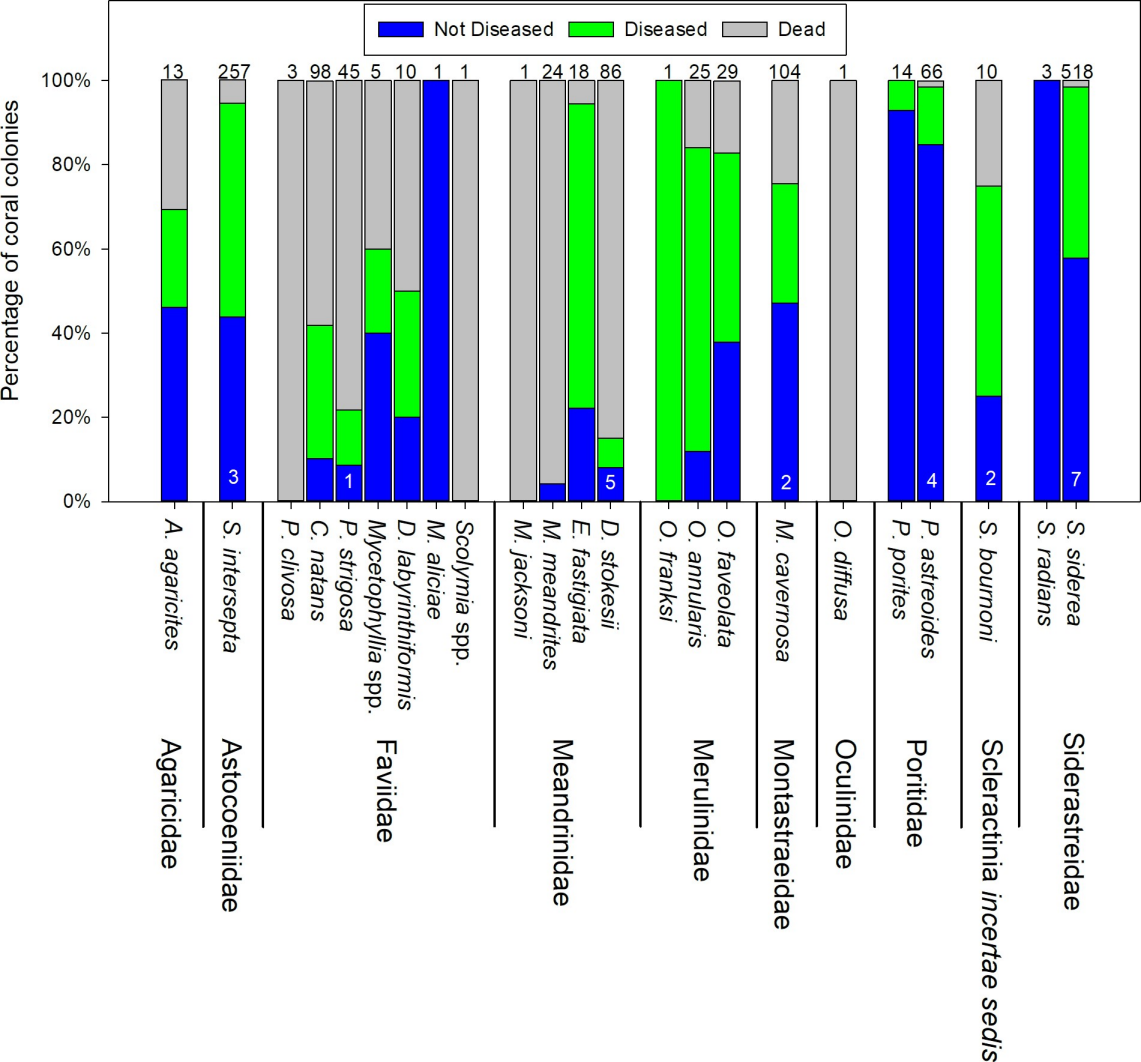
SCTLD status of coral colonies, by species, September 2019. Disease status (Not diseased, diseased, dead) is pooled across sentinel sites. Numbers at the top of the bars represent the number of colonies observed during the September 2019 monitoring period. Numbers at the base of the bars indicate the number of colonies not observed during that monitoring period.

We first observed active SCTLD on February 5, 2018, on an *M*. *meandrites* colony at Grouper Reef. On the next monitoring period, February 16, 2018, SCTLD-affected colonies were observed at Boot Key and Washerwoman Shoal. The first signs of SCTLD at Sombrero Reef were observed during the subsequent monitoring period, March 2, 2018. Evidence of SCTLD was observed through September 2019, with 40–55% of coral colonies across the four sites showing signs of disease and whole colony death per site ranging from approximately 10% to 30% (Fig 5). The mean percentage of living tissue per colony within plots across our four sites decreased from approximately 85–90% in early February to 45–65% by September 2019 (Fig 6A). Nineteen of the 23 coral species exhibited signs of SCTLD (Fig 7). Colonies of species in the Meandrinidae and Faviidae had the greatest proportion of whole colony death, more than 50% of the *C*. *natans*, *Pseudodiploria* spp., *Meandrina* spp., *D*. *stokesii*, and *E*. *fastigiata* colonies experienced 100% colony-level mortality.

We observed a pronounced temporal change in the prevalence of SCTLD during the survey period. The largest proportion of colonies showing signs of SCTLD and the occurrence of whole colony death across all four sites was observed from March to early July 2018 (Figs 5 and 6A). Beginning in mid-July 2018 and continuing through October 2018, new incidence of disease slowed across all four sites, even though colonies of susceptible species remained. The occurrence of whole colony death also slowed (Fig 5). This decrease coincided with seasonal occurrence of coral bleaching during 2018 (Fig 6B). The incidence of bleaching was less severe during 2019, and the decrease in disease-related tissue loss observed during 2018 was not evident.

**Fig 8:**
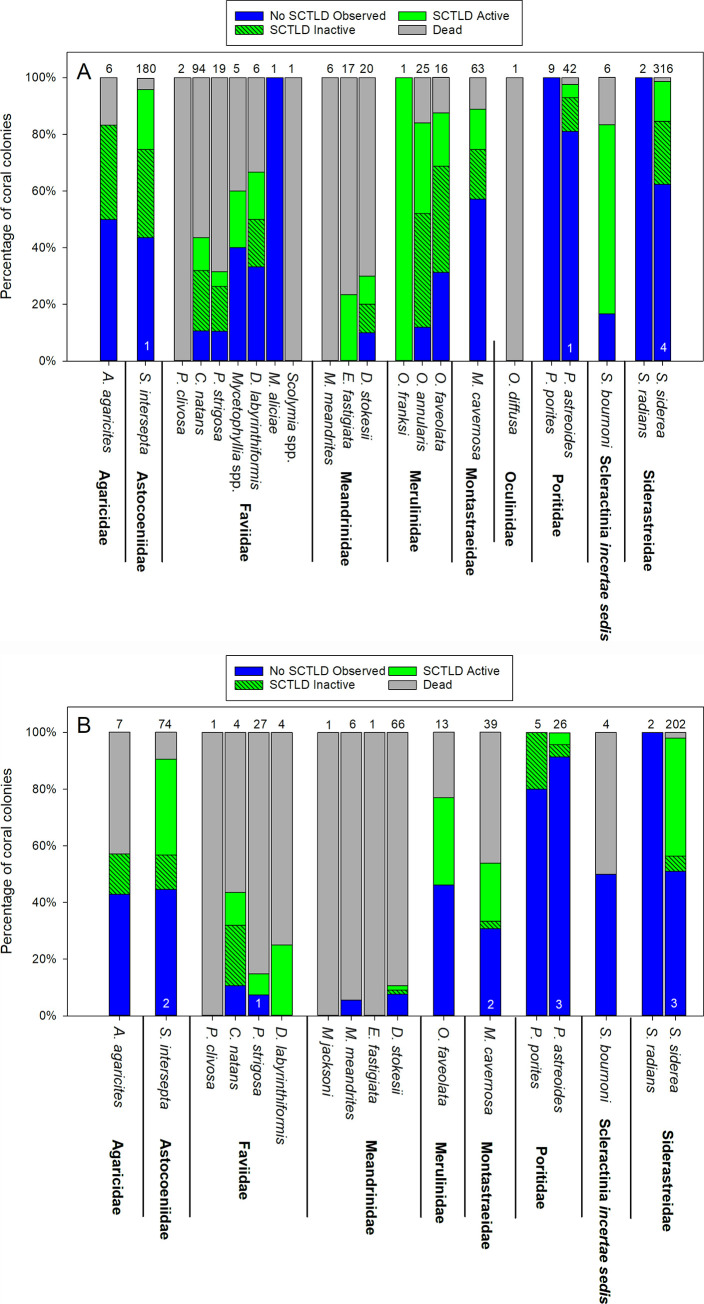
SCTLD status of coral colonies during September 2019, by species. (A) The Boot Key Patch Reef and East Washerwoman Shoal inshore sentinel sites and (B) the Grouper Reef and Sombrero Reef offshore sentinel sites. Diseased colonies defined as SCTLD-inactive are those that showed signs of the disease before July 2018, remained alive, but exhibited no tissue loss after that month. Colonies defined as SCTLD-active were either diseased by July 2018 and continued to lose living tissue or became diseased after July 2018. Numbers at the top of each bar represent the number of colonies observed during the September 2019 monitoring period. Numbers at the base of the bars indicate the number of colonies not observed during that monitoring period.

Beginning with the November 2018 monitoring period and continuing through September 2019, differences in SCTLD prevalence were evident between the inshore and offshore sites. At the two inshore sites, though disease remained present, the number of new colonies becoming diseased among the SCTLD-susceptible colonies and the rate of tissue loss among diseased colonies remained minimal through September 2019, whereas both the number of newly diseased colonies and loss of living tissue increased at the two offshore sites (Fig 5A). In contrast to the offshore sites, many colonies on the inshore sites that had showed signs of disease-related tissue loss during July 2018, including those of the most susceptible species, exhibited no further tissue loss through September 2019 (Fig 8A). The disease remained more active at the two offshore sites, though like the inshore sites, some diseased colonies showed no further tissue loss after July 2018 (Fig 8B).

**Fig 9:**
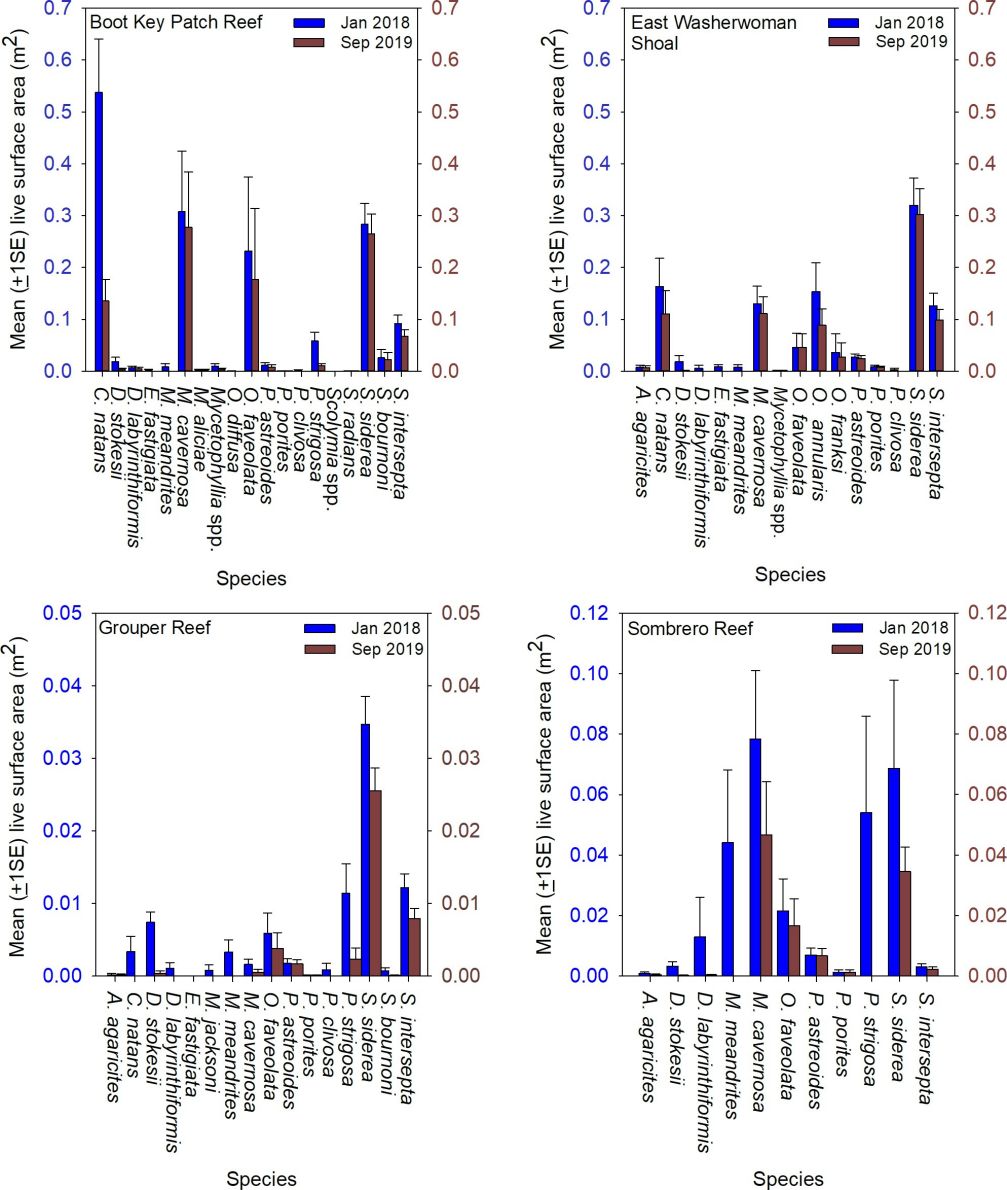
Estimated SA of living coral tissue. The mean (±1SE) living coral tissue (m^2^), per m^2^ by species at each of the four sentinel sites observed during the baseline surveys conducted January 10 through February 2, 2018 and the surveys conducted during September 2019.

Overall, by September 2019 coral community biomass (*i*.*e*. SA of living tissue) had decreased by 39% at Boot Key Patch Reef and by 22% at East Washerwoman Shoal (Fig 9). Coral biomass at these inshore sites remained dominated by *S*. *siderea*, *M*. *cavernosa*, *Orbicella* spp., *S*. *intersepta*, and *C*. *natans*. At the offshore sites, biomass decreased by 50% at Grouper Reef and 62% at Grouper Reef and Sombrero Reef, respectively. Again, all the species that had dominated the community at the offshore reef sites in January 2018 were affected by SCTLD, but little living biomass remained of three of the formerly dominant species, *P*. *strigosa*, *M*. *meandrites*, and *D*. *stokesii*.

### Nearest-neighbor analysis and dynamic multistate occupancy model

#### Multistate occupancy model

We detected anomalous data points in 78 of the 1,355 (5.8%) observations of the surveyed colonies. Those 78 data points included colonies that were either i) not detected during every survey, ii) detected during all surveys but had nonsensical observation histories (e.g., a colony was recorded as showing signs of SCTLD and was subsequently recorded as not showing signs of the disease), or iii) were subject to a combination of both factors. We therefore limited our analysis to only those colonies that were observed during all survey occasions and that had plausible SCTLD status histories. Across all four reefs, with the omission of those 78 colonies, 1,277 colonies with complete observation records were included in the analysis (Boot Key Patch Reef, 418 colonies; Grouper Reef, 307; Sombrero Reef, 154; East Washerwoman Shoal, 398).

The goodness-of-fit assessment indicated that for all four reefs the predicted observations from the fitted model matched the observed data reasonably well, with all Bayesian p-values associated with summaries of the three occupancy states falling within the acceptable range of 0.05–0.95. Additionally, the Gelman-Rubin diagnostic and visual inspection of trace plots indicated no evidence for lack of convergence for any model parameter.

**Fig 10:**
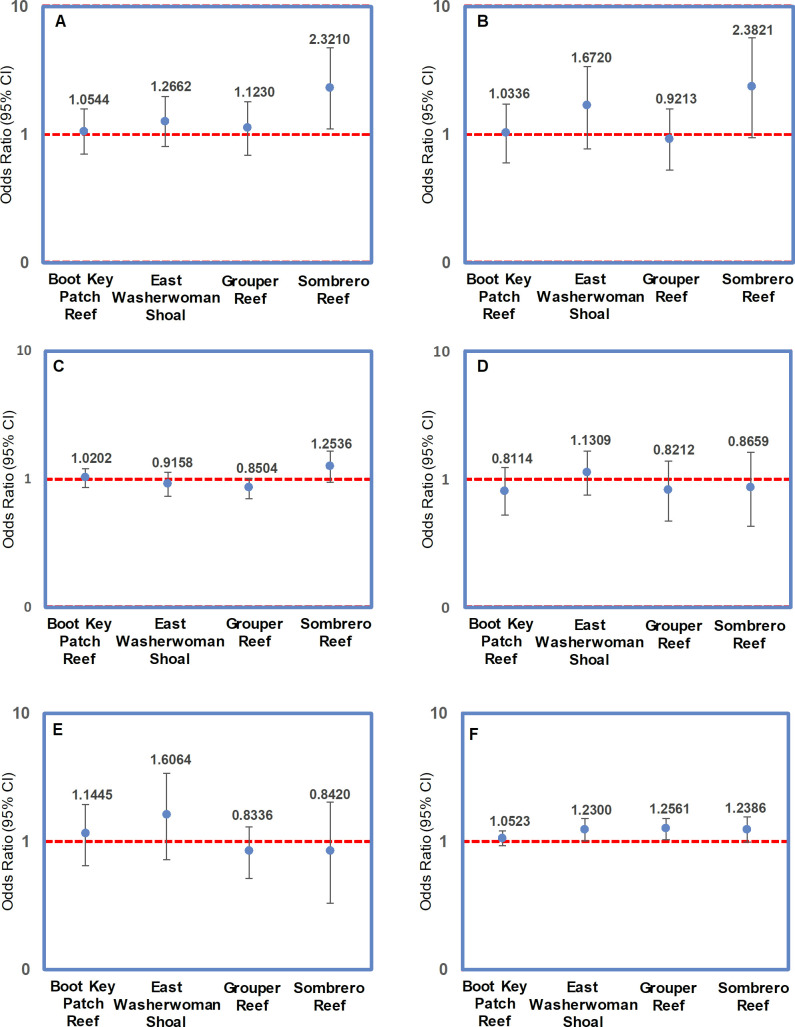
Graphical summation of the odds ratios derived from the transmission model. (A) Odds ratios associated with a non-diseased colony becoming diseased when its nearest neighbor was diseased; (B) Odds ratios associated with a non-diseased colony becoming diseased when its nearest neighbor was dead; (C) Odds ratios associated with a colony becoming diseased in relation to the distance to its nearest neighbor; (D) Odds ratios associated with a non-diseased colony becoming diseased in relation to the distance to its nearest diseased neighbor; (E) Odds ratios associated with a non-diseased colony becoming diseased in relation to the distance to its nearest dead neighbor; (F) Odds ratios associated with a non-diseased colony becoming diseased in relation to its SA.

Overall, the multistate modeling results indicated no conclusive evidence that distance to a colony’s nearest neighbor had any effect on the risk of that colony being affected by SCTLD, regardless of that neighbor’s disease status (results summarized in Table 2). Although there was a positive association at one site (Sombrero Reef; Fig 10A and 10B) between a colony being diseased if its neighbor was either diseased or dead, there was no overall association between disease status and a colony’s distance to its nearest neighboring colony (Fig 10C). Similarly, no association was detected between a colony’s disease status and the distance to its nearest neighboring colony, regardless of the neighbor’s status (Fig 10D and 10E). Modeling results did, however, indicate an association between colony size and the probability of a colony becoming diseased; larger colonies were more likely to be affected by SCTLD at three of the four locations (Grouper Reef, Sombrero Reef, and East Washerwoman Shoal) (Fig 10F).

**Table 2:** Parameter estimates of the dynamic multistate model relating neighbor status and distance to nearest neighbor to the probability of being diseased (i.e., affected by SCTLD).

Parameter	Mean	SD	Lower	Upper	OR	LOR	UOR
**Boot Key Patch Reef**							
Intercept	−3.049	0.57	−4.118	−1.886			
Nearest neighbor Diseased	0.053	0.203	−0.350	0.445	1.054	0.705	1.560
Nearest neighbor Dead	0.033	0.266	−0.503	0.544	1.034	0.605	1.723
Distance to nearest colony	0.02	0.082	−0.144	0.181	1.020	0.866	1.198
Colony surface area	0.051	0.068	−0.092	0.178	1.052	0.912	1.195
Distance × Diseased	−0.209	0.213	−0.64	0.198	0.811	0.527	1.219
Distance × Dead	0.135	0.283	−0.438	0.662	1.145	0.645	1.939
**East Washerwoman Shoal**							
Intercept	−3.079	0.676	−4.173	−1.419			
Nearest neighbor Diseased	0.236	0.225	−0.219	0.670	1.266	0.803	1.954
Nearest neighbor Dead	0.514	0.379	−0.256	1.225	1.672	0.774	3.404
Distance to nearest colony	−0.088	0.107	−0.304	0.117	0.916	0.738	1.124
Colony surface area	0.207	0.102	0.003	0.401	1.23	1.003	1.493
Distance × Diseased	0.123	0.198	−0.277	0.502	1.131	0.758	1.652
Distance × Dead	0.474	0.39	−0.318	1.216	1.606	0.728	3.374
**Grouper Reef**							
Intercept	−2.887	0.509	−3.831	−1.845			
Nearest neighbor Diseased	0.116	0.244	−0.372	0.582	1.123	0.689	1.790
Nearest neighbor Dead	−0.082	0.279	−0.643	0.453	0.921	0.526	1.573
Distance to nearest colony	−0.162	0.092	−0.348	0.012	0.850	0.706	1.012
Colony surface area	0.228	0.096	0.035	0.412	1.256	1.036	1.510
Distance × Diseased	−0.197	0.277	−0.762	0.326	0.821	0.467	1.385
Distance × Dead	−0.182	0.230	−0.659	0.255	0.834	0.517	1.290
**Sombrero Reef**							
Intercept	−2.096	0.81	−3.523	−0.368			
Nearest neighbor Diseased	0.842	0.372	0.088	1.546	2.321	1.092	4.693
Nearest neighbor Dead	0.868	0.457	−0.056	1.724	2.382	0.946	5.607
Distance to nearest colony	0.226	0.141	−0.057	0.492	1.254	0.945	1.636
Colony surface area	0.214	0.113	−0.017	0.428	1.239	0.983	1.534
Distance × Diseased	−0.144	0.343	−0.856	0.482	0.866	0.425	1.619
Distance × Dead	−0.172	0.460	−1.118	0.702	0.842	0.327	2.018

Parameters summarized are: mean (Mean), standard deviations (SD), lower 95% confidence limit (Lower), upper 95% confidence limit (Upper), odds ratios (OR), and lower and upper 95% confidence limits of the odds ratios (LOR and UOR). Coefficients associated with colony surface area and distance to nearest neighbor represent the change in log odds of being diseased for every increase of 1 standard deviation in the predictor variable (Surface area, cm^2^: Boot Key, mean = 1,182.12, sd = 3,490.06; Grouper Reef, mean = 357.63, sd = 448.18; Sombrero Reef, mean = 1,532.82, sd = 3,709.95; East Washerwoman Shoal, mean = 875.61, sd = 1,633.55. distance to nearest neighbor, cm: Boot Key, mean = 17.26, sd = 9.69; Grouper Reef, mean = 39.88, sd = 28.14; Sombrero Reef, mean = 40.75, sd = 29.40; East Washerwoman Shoal, mean = 18.67, sd = 10.26). Note that model intercepts associated with each reef represent the mean log-odds of infection for a colony with average SA at an average distance from its nearest neighbor (due to standardizing of continuous covariates), averaged across time periods and species (due to species- and time-specific random effects).

## Discussion

Our high-frequency surveys have documented the first description of which we are aware of the initial appearance of SCTLD and its progression through previously unaffected coral communities and temporal changes in its transmission rate. Although the etiology of SCTLD remains unresolved, and our monitoring documented only tissue loss in coral colonies that was presumably related to SCTLD, the expression of the affected colonies we documented was largely consistent with the current case descriptions of its epidemiology [12, 13, 15, 30]. The disease transmission encompassed a wide range of species, with affected colonies typically exhibiting single or multifocal lesions of newly exposed, bright-white coral skeleton, indicating rapidly progressing tissue mortality but with clearly different susceptibilities to the disease among species, with species belonging to the families Meandrinidae and Faviidae being especially susceptible, commonly resulting in whole colony death.

Previous observations of cross-reef transmission at the onset of SCTLD suggest that the associated pathogens are waterborne, with coastal currents likely the primary driver of its spread [9, 12], and our results are consistent with this hypothesis. After the first observation of SCTLD at one of our offshore sites, the disease was observed within two weeks at the other offshore site and had progressed several km across the reef to both inshore sites. Moreover, our multistate occupancy model indicated little evidence of an association between the disease state of, and distance to, its nearest neighboring colony. Combined, these findings suggest that disease transmission was independent of coral colony density. Finally, the model indicated that the probability of a colony becoming affected by SCTLD increased with increasing colony SA, a relationship previously documented in infections by other waterborne coral epizootics [31–33]. Those studies speculated that the combined effects of greater SA results in increased exposure to waterborne pathogens, causing multiple, presumably independent, disease lesions in the same colony. Although we did not record the number of lesions on diseased colonies, lesions were commonly multifocal and, at least externally, seemingly independent of each other.

We cannot, however, discount the possibility that transmission could also be facilitated by other vectors and direct contact, and indeed could represent an important mode of transmission at small-scales over longer time-frames, particularly in areas where SCTLD persists chronically at low levels. For example, corallivorous gastropods, polychaetes, and fishes, particularly Chaetodontids, have been implicated as active vectors of coral disease [6, 7, 34], as has physical contact with certain macroalgae [35]. Additionally, there is evidence that Rhodobacterales and Rhizobiales transmitted via sediment are associated with SCTLD [36]. However, if factors other than–or in addition to–waterborne transmission contributed to the spread of SCTLD, we suspect that the rate at which SCTLD spread through our sites could have obscured their roles, even as the rates of SCTLD decreased later in our survey period.

It is intriguing that the rate of tissue loss among SCTLD-affected colonies and the occurrence of SCTLD on previously unaffected colonies showing the initial signs of SCTLD slowed or stopped across all the sentinel sites during the summer of 2018, coinciding with the seasonal peak in coral bleaching. It is reasonably well documented that coral colonies compromised by bleaching are associated with disease [9, 37–40]. The initial reports of SCTLD noted the association between the onset of SCTLD in the upper Florida Keys and elevated summer water temperatures and bleaching [12], though broader-scale epidemiological modeling did not detect an association with SCTLD and sea surface temperature [9]. We did not observe a similar decrease in disease rates during the summer of 2019, when the incidence of bleaching was comparatively less severe than in 2018. Therefore, it bears speculation that SCTLD susceptibility may be driven in part by a pathogen-coral-endosymbiont association. The functional relationship between disease incidence in corals is not well resolved, but there is evidence suggesting that functional differences among algal symbionts affect disease susceptibility in some corals, however, this relationship is complex as endosymbiont communities vary among coral species [41–43]. Alternatively, we note that preliminary histological assessments suggest that the external lesions that typify SCTLD are expressed in the later stages of the colony becoming affected, and consequently our visual assessments may not truly reflect temporal trends in early-stage SCTLD prevalence [44]. We have continued to monitor our sites and will be modeling temporal- and species-specific tissue loss rates of affected colonies to inform the broader SCTLD response effort to better resolve possible seasonally-mediated disease dynamics.

Through the initial months of our survey there was little difference between sites as SCTLD rapidly spread. However, after the summer 2018 bleaching event had abated, discernable differences in SCTLD progression rates between the inshore and offshore sites were evident. Notably, SCTLD incidence remained lower at the higher density inshore sites, contrary to reports elsewhere that have documented an association between disease incidence and high colony density [40, 45]. However, given the generalist nature exhibited by SCTLD [11] and again, the absence of nearest neighboring effects (e.g. clustering) it is not surprising that it does not follow this density-disease relationship.

The differential prevalence of SCTLD between the inshore and offshore coral communities is consistent with previous observations on the FRT. In the upper Florida Keys, a study that monitored SCTLD prevalence in two species, *P*. *strigosa* and *S*. *siderea* located at nearshore patch reefs and offshore reefs documented a higher incidence of colonies exhibiting SCTLD offshore than at inshore locations [46]. There is a wealth of evidence demonstrating differential resistance to environmental stress among certain coral colonies, and that coral communities in the comparatively more environmentally dynamic inshore locations, including those along the FRT, are more resilient to stressors than offshore communities [47–51]. The mechanisms underlying these localized adaptations are not well understood due to the complex mutualistic interactions of the coral holobiont with the environment. Resilience could lie in coral genotype itself, as there is evidence that certain coral communities may dynamically regulate gene expression in response to stress and adapted to exhibit innate immune responses to disease [51, now 52], or with their associated endosymbionts [53–56]. Thus, it bears speculation that localized adaptation among the inshore coral communities may confer some greater degree of resilience to SCTLD.

There is a coordinated effort by resource managers and researchers in Florida to address the SCTLD epizootic. This response is composed of a multi-pronged strategy that includes: i) the development of direct intervention techniques aimed at mitigating SCTLD mortality by applying antiseptics or an antibiotic directly to SCTLD lesions on affected colonies; ii) a “coral rescue” effort to collect disease-free colonies of SCTLD susceptible species from the FRT and maintain and spawn them within land-based facilities with the vision of eventually reintroducing them to the wild once the threat of SCTLD has diminished; and iii) a coordinated effort to investigate the histology, microbiology, and spatial epidemiology of SCTLD [57]. The primary goal of our high-frequency monitoring effort was to assess the small-scale spatial epidemiology and temporal transmission rates specifically to inform both the intervention and rescue efforts. The apparent greater resistance of inshore coral communities to SCTLD relative to those offshore, even for species clearly more susceptible to the disease, should prove of interest in guiding the coral rescue and land-based captive spawning effort. These colonies may represent particularly resilient genotypes or holobiont community. Identifying such colonies and ensuring that they are incorporated into this rescue effort merits consideration.

The intervention efforts under way along the FRT have had some success in slowing and stopping the progression of SCTLD lesions in affected colonies [18], though our results underscore that, given its waterborne mode of transmission, presumably via both along- and cross-reef currents, SCTLD can simultaneously affect an area of the reef tract that comprises thousands of coral colonies. Consequently, when SCTLD is simultaneously affecting the number of colonies that we documented early in our study, these efforts will have limited success in mitigating its transmission throughout the neighboring coral community. Moreover, despite the success of directly treating lesions on affected colonies, additional—and presumably independent—lesions can still occur in the treated colonies [58]. Refinements in such techniques, however, may yet prove a successful strategy to save individual coral colonies and their reproductive potential, especially large, slow-growing colonies [59].

Finally, although our monitoring effort provided a detailed evaluation of the mode and speed of progress at the colony and cross-reef scale, further spatial epidemiological work incorporating hydrographic modeling is needed. These models should include the movement of oceanographic currents over realistic seafloor topography to provide the Caribbean region’s conservation managers with better understanding of how SCTLD moves at larger scales and enable them to develop and implement tractable responses to this event.

## Supporting information

S1 Material (CSV)

S2 Material (XLSX)
